# FAMP, a Novel ApoA‐I Mimetic Peptide, Suppresses Aortic Plaque Formation Through Promotion of Biological HDL Function in ApoE‐Deficient Mice

**DOI:** 10.1161/JAHA.113.000048

**Published:** 2013-06-21

**Authors:** Yoshinari Uehara, Setsuko Ando, Eiji Yahiro, Kosuke Oniki, Makoto Ayaori, Satomi Abe, Emi Kawachi, Bo Zhang, Seijiro Shioi, Hiroyuki Tanigawa, Satoshi Imaizumi, Shin‐ichiro Miura, Keijiro Saku

**Affiliations:** 1 Department of Cardiology, Fukuoka University, Japan (Y.U., E.Y., K.O., S.A., E.K., H.T., S.I., S.M., K.S.); 2 Department of Molecular Cardiovascular Therapeutics, Fukuoka University, Japan (Y.U., S.I., S.M., K.S.); 3 Department of Chemistry, Faculty of Science, Fukuoka University, Japan (S.A.); 4 Division of Anti‐aging and Vascular Medicine, Department of Internal Medicine, National Defense Medical College, Japan (M.A.); 5 Department of Advenced Therapeutics for Cardiovascular Disease, Fukuoka University, Japan (S.A., H.T., K.S.); 6 Department of Biochemistry, Fukuoka University School of Medicine, Japan (B.Z.); 7 Radioisotope Center, Fukuoka University, Japan (S.S.)

**Keywords:** apolipoproteins, ATP‐binding cassette transporters, HDL particle size, peptides, pre‐β HDL

## Abstract

**Background:**

Apolipoprotein (apo) A‐I is a major high‐density lipoprotein (HDL) protein that causes cholesterol efflux from peripheral cells through the ATP‐binding cassette transporter A1 (ABCA1), thus generating HDL and reversing the macrophage foam cell phenotype. Pre‐β_1_ HDL is the smallest subfraction of HDL, which is believed to represent newly formed HDL, and it is the most active acceptor of free cholesterol. Furthermore it has a possible protective function against cardiovascular disease (CVD). We developed a novel apoA‐I mimetic peptide without phospholipids (Fukuoka University ApoA‐I Mimetic Peptide, FAMP).

**Methods and Results:**

FAMP type 5 (FAMP5) had a high capacity for cholesterol efflux from A172 cells and mouse and human macrophages in vitro, and the efflux was mainly dependent on ABCA1 transporter. Incubation of FAMP5 with human HDL or whole plasma generated small HDL particles, and charged apoA‐I‐rich particles migrated as pre‐β HDL on agarose gel electrophoresis. Sixteen weeks of treatment with FAMP5 significantly suppressed aortic plaque formation (scrambled FAMP, 31.3±8.9% versus high‐dose FAMP5, 16.2±5.0%; *P*<0.01) and plasma C‐reactive protein and monocyte chemoattractant protein‐1 in apoE‐deficient mice fed a high‐fat diet. In addition, it significantly enhanced HDL‐mediated cholesterol efflux capacity from the mice.

**Conclusions:**

A newly developed apoA‐I mimetic peptide, FAMP, has an antiatherosclerotic effect through the enhancement of the biological function of HDL. FAMP may have significant atheroprotective potential and prove to be a new therapeutic tool for CVD.

## Introduction

Various clinical and epidemiological studies have demonstrated a negative correlation between high‐density lipoprotein (HDL) cholesterol levels and the risk of cardiovascular events and shown that HDL exerts many potential antiatherogenic effects.^1^ For example, HDL particles transport cholesterol from peripheral cells to the liver and steroidogenic organs, where cholesterol is used for synthesis of bile acids, lipoproteins, vitamins, and steroid hormones.^1^ Apolipoprotein (apo) A‐I is a major HDL protein, and both lipid‐poor apoA‐I and discoidal HDL have pre‐β electrophoretic mobility (pre‐β HDL), unlike the majority of HDL particles migrating with alpha electrophoretic mobility (α HDL) in agarose gel electrophoresis.^2–3^ Previous studies have demonstrated that the serum pre‐β HDL fraction represents 1% to 25% of total apoA‐I in humans.^4–6^

Pre‐β_1_ HDL, the smallest subfraction of HDL comprising small amounts of phospholipids and apoA‐I, is believed to represent nascent or newly formed HDL.^7–8^ Experimental studies have demonstrated that pre‐β HDL is the most active acceptor of free cholesterol both in vitro and in vivo.^9–10^ Several studies have demonstrated a positive correlation between serum pre‐β_1_ HDL level and ATP‐binding cassette transporter (ABC) A1‐mediated efflux capacity^11^ as well as decreased plasma pre‐β_1_ HDL levels in patients with coronary heart disease (CHD).^12^ Other studies have associated increased pre‐β_1_ HDL levels with exercise^13^ and fibrate treatment,^14^ suggesting a possible protective function of pre‐β_1_ HDL against CHD. However, these promising results have been controversial because higher pre‐β_1_ HDL levels are generally observed in patients with CHD who have relatively low HDL cholesterol levels.^15–16^

*ABCA1* has been identified as a pivotal gene in the regulation of plasma HDL cholesterol levels and cellular cholesterol homeostasis, and it is defective in Tangier disease patients, an HDL deficiency.^17–19^ The interaction between ABCA1 and apoA‐I leads to the lipidation of apoA‐I, which then forms the nascent pre‐β (pre‐β_1_) HDL particle, an important initial step for reverse cholesterol transport.^20^

As a therapeutic approach for increasing HDL, many researchers have focused on not only increasing HDL cholesterol levels but also enhancing its biochemical functioning. HDL therapies using injections of reconstituted HDL, apoA‐I mimetics, or full‐length apoA‐I have been dramatically effective.^21–22^ Nissen et al^22^ showed that in humans ETC‐216, an apoA‐I‐Milano complexed with phospholipids and intravenously administered, produced a significant regression of coronary atherosclerotic plaque as measured by intravascular ultrasound (IVUS). After infusion of ETC‐216, regression of coronary atherosclerosis was accompanied by reverse remodeling of the external elastic membrane, with no changes in the luminal dimensions as assessed by IVUS.^23^ These results support the idea that short‐term infusions are rapidly effective for preventing the progression of atherosclerosis. Although studies on the use of apoA‐I mimetic peptides (4F and L37pA, etc) are being conducted,^24–26^ none are currently available for clinical use. To develop a physiological HDL‐generating apoA‐I mimetic peptide worked with ABCA1 transporter, various candidate peptides were synthesized that focused on the amino acid sequence alignments of human apoA‐I interacting with ABCA1. Here we developed a novel short apoA‐I mimetic peptide consisting of 24 amino acids and without phospholipids (Fukuoka University ApoA‐I Mimetic Peptide [FAMP]), which retains the amphipathic helical structure of the 243–amino acid apoA‐I and its ability to associate with lipids. In addition, promotion of biological HDL function and antiatherosclerotic action of the developed peptide were elucidated.

## Methods

### Reagents and Antibodies

Human apoA‐I, HDL, and endotoxin‐free bovine serum albumin (BSA) were purchased from Calbiochem. Probucol, 8Br‐cAMP, the synthetic liver X receptor (LXR) agonist T0901317, and retinoid X receptor (RXR) ligands 9‐*cis*‐retinoic acid were purchased from Sigma. Fmoc‐amino acids and coupling reagents were purchased from Watanabe Chemical Ind. Ltd. A goat polyclonal antihuman apoA‐I antibody was purchased from Sekisui Medical Co, Ltd.

### Peptide Synthesis

ApoA‐I fragments, FAMPs, scrambled FAMP, and acridone (ACD)‐FAMP were synthesized by Fmoc (*N*‐[9‐fluorenyl] methoxycarbonyl)–based solid‐phase peptide synthesis using an automated peptide synthesizer, Pioneer and Model 433A, from Applied Biosystems, Inc, employing the standard Fmoc methodology as described previously.^27^ Piperidine/*N*‐methylpyrrolidone (NMP; 20%) was used as a deblock reagent, and 2‐(1 H‐benzotriazol‐1‐yl)—1,1,3,3‐tetramethyluronium hexafluorophosphate—and 1‐hydroxybenzotriazole were used as coupling reagents. The peptides were cleaved from the resin with an ethanedithiol/H_2_O/trifluoroacetic acid (TFA) mixture (0.25/0.25/9.5 *v*/*v*/*v*). Cold ether was used to crystallize the peptides after removing TFA using N_2_ gas. The obtained crude peptides were passed through a Sephadex G‐25 gel filtration chromatograph. Then the peptides were purified by a preparative Inertsil ODS‐3 high‐performance liquid chromatography (HPLC) column (20×250 mm; GLSience). The purity of the final products was evaluated by analytical reverse‐phase HPLC and was found to exceed 95%. Matrix‐assisted laser desorption/ionization‐time‐of‐flight mass analysis was performed using a Perseptive BioSystems VOYAGER Delayed Extraction (DE‐STR) spectrometer and sinapic acid as a matrix. The circular dichroism (CD) spectra of the peptides were recorded on a JASCO J‐720 spectropolarimeter with a thermostatic cell holder using a quartz cell with a path length of 0.1 mm on a wavelength range of 200 to 260 nm. The peptides and human apoA‐I were dissolved in the Tris‐HCl buffer to a concentration of 17.8 to 178 μmol/L at 25°C. Mean residue ellipticity was given as deg cm^2^ dmol^−1^.

### Cell Preparations and Cultures

A172 human glioblastoma, COS‐7 cells (Health Science Research Resources Bank, Osaka, Japan), and RAW264 mouse macrophage‐like cells (RIKEN BRC) were cultured in DMEM containing 10% fetal bovine serum (Life Technologies Japan Ltd), 100 units/mL penicillin G, and 100 μg/mL streptomycin. Peritoneal macrophages were obtained from mice injected intraperitoneally with 2 mL of 2.9% thioglycollate (Nissui Pharmaceutical Co) 90 hours before the experiment and prepared as adherent cells by incubating for 2 hours. Peripheral blood monocyte‐derived macrophages were isolated using Lymphoprep Separation Solution (Nycomed Pharma) as described previously.^28^ For all experiments, cells were maintained in serum‐free medium containing 0.2% BSA with or without additives (5 μmol/L T0901317 and 9‐*cis*‐retinoic acid, 10 μmol/L probucol, or 0.3 mmol/L 8Br‐cAMP).

### Plasmid Constructs and Transient Transfection

Wild‐type human *ABCA1* and *ABCG1* cDNA was subcloned into the pcDNA3.1 and pEdsRed‐N1 vectors, respectively. Chinese hamster ovary (CHO)‐ldlA7^29^ and COS‐7 cells were seeded at 80% to 90% confluence in 24‐well dishes and transfected using Lipofectamine 2000 reagent (Invitrogen) as described previously.^30^

### Cellular Cholesterol Efflux

A172 human glioblastomas, CHO‐ldlA7 cells transiently transfected with human *ABCA1* cDNA, COS‐7 cells transiently transfected with human *ABCA1* and *ABCG1* cDNAs, human blood monocyte‐derived macrophages, RAW264 murine macrophages, and murine peritoneal macrophages were used for cholesterol efflux experiments. The cells were radiolabeled with ^3^H‐cholesterol, and cellular cholesterol efflux was measured as described previously.^28,31^

### Hemolysis Assays

Erythrocytes were collected from EDTA‐treated human blood from 4 individual subjects by centrifugation and then washed 3 times with phosphate buffered saline to remove plasma and buffy coat. A suspension of 1% erythrocytes in phosphate‐buffered saline with or without peptide (1 to 1000 μg/L for final concentration) was incubated for 10 minutes at 37°C. Hemolysis was measured as described previously.^32^ Hemolysis was expressed as a percentage of the Triton X‐100 lysis.

### Lipoprotein Analyses by Agarose Gel Electrophoresis and ApoA‐I Immunoblotting

Ηuman plasma samples were incubated with FAMP5, ACD‐FAMP5, or saline at 37°C for 60 minutes. Agarose gel electrophoresis and differential staining were performed using a Rapid Electrophoresis System (REP, Helena Laboratories) according to the method described previously.^33^ REP Lipo‐30 plates and CHOL/TRIG COMBO (CH; KK Helena Kenkyujo) were used as the agarose gel and reagents, respectively, for staining of cholesterol but not phospholipids and triglycerides. After transfer to a PVDF membrane, apoA‐I was identified by immunoblotting with antihuman apoA‐I antibody. The samples incubated with ACD‐FAMP5 were detected by fluorescent signaling on the agarose gel under UV light.

### Size Analysis of HDL Interacting With FAMP

HDL was adjusted to 2.22 mg/mL with saline containing 0.1% EDTA (pH 7.4). The samples were incubated with final concentrations of 50, 250, or 500 μg/mL FAMP5 for 60 minutes. Loading buffer consisting of 0.1% bromophenol blue in 1 mol/L Tris‐HCl (pH 6.8)/glycerol/dH_2_O (3.125/5/1.875 *v*/*v*/*v*) was added to the samples at a final concentration of 20% (*v*/*v*). HDL subclasses in the sample were separated by their hydrodynamic diameter in a nondenaturing 5% to 20% gradient polyacrylamide gel containing Tris‐glycine buffer (pH 7.5) for 3 hours at 20 mA in an ice bath. The electrophoresis gels were stained for proteins with Coomassie Brilliant Blue (CBB) R‐250. Reference globular proteins (thyroglobulin, 17 nm; ferritin, 12.2 nm; catalase, 9.2 nm; lactate dehydrogenase, 8.2 nm; and albumin, 7.1 nm) were run in the same gels as markers.

### Mice

The study protocol was approved by the Animal Care and Use Committee of Fukuoka University. Seven‐week‐old male C57BL6 mice were purchased from KBT Oriental (Tokyo, Japan) and housed in specific pathogen‐free barrier facilities at Fukuoka University. Six‐week‐old male apoE‐deficient spontaneously hyperlipidemic mice (C57BL/6.KOR/StmSlc‐*Apoe*^*shl*^; apoE^−/−^) were purchased from SLC Co Ltd. Mice were maintained under a 12‐hour light/dark cycle, fed a standard rodent diet (CLEA Japan at Nihon Bioresearch), and provided with water ad libitum except where noted.

### Pharmacokinetics of FAMP5 in C57BL6 and ApoE^−/−^ Mice

Both C57BL6 (n=3) and apoE‐deficient mice (n=3) were intravenously administered 10 mg/kg of fluorescence‐labeled ACD‐FAMP5. Plasma fluorescence signals were measured 0, 1, 5, 10, 15, 30, 60, 90, 120, 180, 240, 360, 480, 720, 1440, and 2880 minutes after ACD‐FAMP5 injection. Fractional catabolic rate (FCR) was calculated from the fitted decay curve by an in‐house program made using the SAS (Statistical Analysis System) software package (Ver. 9.2; SAS Institute Inc) at Fukuoka University.

### Single Dose of FAMP Treatment in ApoE^−/−^ Mice

C57BL6 and apoE^−/−^ mice were intraperitoneally injected with saline or ACD‐FAMP5 (50 mg/kg of body weight). After 24 hours, overnight fasting plasma samples were collected from the mice treated with saline or ACD‐FAMP5.

### Analyses of Lipoprotein Profiles by Fast Protein Liquid Chromatography

For lipoprotein fractionation analyses, equal volumes of plasma samples were pooled from mice of each group (total volume, 400 μL). Lipoproteins were fractionated by fast protein liquid chromatography (FPLC) using a Superose 6 10/300 GL FPLC column (Amersham Biosciences). Fractions (500 μL) were collected and used for cholesterol measurements.

### Sixteen‐Week Treatment With FAMP in ApoE^−/−^ Mice

ApoE^−/−^ mice (n=23) fed a high‐fat diet (0.5% cholesterol and 10% fat) were intraperitoneally administered either a high dose (50 mg/kg of body weight per day) or low dose (10 mg/kg of body weight per day) of FAMP5 or scrambled FAMP 3 times per week for 16 weeks. After 16 weeks, overnight fasting plasma samples were collected from 19 of the 23 apoE^−/−^ mice treated with scrambled FAMP or FAMP5.

### Evaluation of Aortic Atherosclerotic Lesion Formation

After the 16‐week treatment with FAMP5 or scrambled FAMP, whole aortas were collected, and the extracted aortic tissues were stained with Oil Red O. Oil Red O–stained plaque lesions were calculated using ImageJ 1.45s software, and the extent of atherosclerosis was expressed as the percentage of the lesion area extending from the ascending aorta to the abdominal bifurcation.

### *Ex Vivo* Cholesterol Efflux

After 16 weeks, plasma samples were collected from the mice. The plasma HDL (apoB‐depleted plasma)–mediated cholesterol efflux was measured in 8Br‐cAMP‐treated RAW264 macrophages as described above.

### Analyses of Lipoprotein Profiles by HPLC

After 16 weeks, the collected plasma lipoprotein profiles were analyzed by HPLC at Skylight Biotech Inc according to the procedure described by Usui et al.^34–35^

### Measurement of Plasma Inflammation Markers

Plasma high‐sensitive C‐reactive protein (CRP) levels from apoE^−/−^ mice were measured using an ELISA assay kit purchased from KAMIYA Biomedical Company. Mouse plasma interleukin 6 (IL‐6) and monocyte chemoattractant protein‐1 (MCP‐1) levels were measured using an ELISA assay kit (R&D Systems, Inc).

### Statistical Analysis

All statistical analyses were performed using SigmaStat software (SYSTAT Software Inc, San Jose, CA). Differences between groups were analyzed by an unpaired *t* test or 1‐way analysis of variance followed by Fisher's protected least significant difference, and data are expressed as mean±SD.

### Plasma Samples and Peripheral Blood Monocyte‐Derived Macrophages From Healthy Volunteer and TD Patients

This study was approved by the local ethics committee of Fukuoka University (No. 064), and written consent was obtained from all subjects.

## Results

### Synthetic Peptide Fragments of Human ApoA‐I and FAMPs

**Figure 1.**
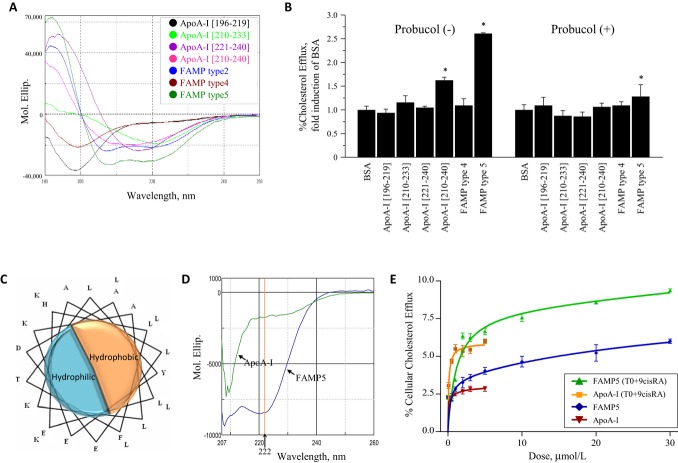
Characteristics of human apoA‐I fragments and apoA‐I mimetic peptides. A, Circular dichroism (CD) spectra of human apoA‐I fragments and FAMP5 in water. The apoA‐I peptide fragment consisting of amino acids 210 to 240 was the only fragment in the region of apoA‐I that formed an α‐helical conformation. FAMP2 and FAMP5, but not FAMP4, formed strong α‐helical conformations. B, Effects of various human apoA‐I fragments and FAMPs on cellular cholesterol efflux in A172 human cells. Cellular cholesterol efflux mediated by apoA‐I fragments and FAMP4 and FAMP5 were measured in human A172 cells with or without probucol (10 μmol/L) in the presence of T0901317 (5 μmol/L) and 9‐*cis*‐retinoic acid (9*cis*RA; 5 μmol/L). The experiments were performed for 4 hours in the presence or absence of 20 μg/mL apoA‐I fragments, or FAMPs (n=3 to 5 for each group). C, Helix wheel representation of FAMP5. D, CD spectra of human apoA‐I and FAMP5 in buffer with 3 mol/L guanidine hydrochloride, 10 mmol/L Tris, and 5 mmol/L dithiothreitol (DTT). E, Dose‐dependent increases on a molar basis in human apoA‐I‐ and FAMP5‐mediated cellular cholesterol efflux for 4 hours are shown in the presence or absence of 5 μmol/L of T0901317 or 9‐*cis*‐retinoic acid in A172 cells (n=4 each). Values are mean±SD. *P<0.01 vs BSA group. FAMP indicates Fukuoka University apoA‐I mimetic peptide; apoA‐I, apolipoprotein A‐I; BSA, bovine serum albumin.

Several candidate peptide fragments were synthesized on the basis of the amino acid sequence alignments with human apoA‐I (Table 1). Based on previous findings,^36–39^ we synthesized apoA‐I mimetic peptides corresponding to amino acids 220 to 231 of the C‐terminus of human apoA‐I. Unfortunately, these peptides did not have α‐helical conformations (Figure 1A). Furthermore, neither cellular cholesterol efflux nor α‐helical conformation was observed with peptides of 24 amino acids or less, corresponding to amino acids 196 to 240 of human apoA‐I (Figure 1B). Nevertheless, the 31–amino acid peptide corresponding to residues 210 to 240 of human apoA‐I had an α‐helical conformation (Figure 1A). Because the 24‐residue peptide corresponding to amino acids 196 to 219 of human apoA‐I acquired an α‐helical structure, several novel peptides were synthesized with replacements of 3 to 8 of the 24 amino acids in this region (Table 1). FAMP2 and FAMP5, but not FAMP4, formed strong α‐helical conformations (Figure 1A). The FAMP5 peptide, which was 67% homologous to the region of amino acids 196 to 219 of human apoA‐I, formed a typical α‐helical conformation and had amphipathic helix potential in solution despite being only 24 amino acids (Figure 1A and 1C). We also examined the CD spectra of FAMP5 compared with human apoA‐I in the same buffer (Figure 1D). The alpha‐helicity of FAMP5 and human apoA‐I was estimated to be 29.3% and 12.5%, respectively, at a wavelength of 222 nm, and the ellipticity at 222 nm indicated that FAMP5 was 4.7‐fold higher in alpha‐helicity compared with human apoA‐I.

**Table 1.** Amino Acid Alignments of Human ApoA‐I Fragments and ApoA‐I Mimetic Peptides

Name	Amino Acid Sequence	Number of Amino Acids
ApoA‐I (196 to 219)	H‐ATEHLSTLSEKAKPALEDLRQGLL‐OH	24
ApoA‐I (210 to 233)	H‐ALEDLRQGLLPVLESFKVSFLSAL‐OH	24
ApoA‐I (221 to 240)	H‐VLESFKVSFLSALEEYTKKL‐OH	20
ApoA‐I (210 to 240)	H‐ALEDLRQGLLPVLESFKVSFLSALEEYTKKL‐OH	31
FAMP type 1 (FAMP1)	H‐ALEHLFTLSEKAKKALEDLLKKLL‐OH	24
FAMP type 2 (FAMP2)	H‐ALEHLFTLSEKALKALEDLLKKLL‐OH	24
FAMP type 3 (FAMP3)	H‐ATEHLSTLSEKALKAFEDLLKKLL‐OH	24
FAMP type 4 (FAMP4)	H‐ATEHLSTLSEKAKPALEDLLKKLL‐OH	24
FAMP type 5 (FAMP5)	H‐ALEHLFTLYEKALKALEDLLKKLL‐OH	24
FAMP type 6 (FAMP6)	H‐ALEHLFTLWEKALKALEDLLKKLL‐OH	24
FAMP type 12 (FAMP12)	H‐YALEHLFTLSEKALKALEDLLKKLL‐OH	25
Scrambled FAMP	H‐ATEFLHLLLLKLLKKKEDELLAYA‐OH	24
Ac‐FAMP5‐NH_2_	Ac‐ALEHLFTLYEKALKALEDLLKKLL‐NH_2_	24
L‐4F	Ac‐DWFKAFYDKVAEKFKEAF‐NH_2_	18
H‐L‐4F‐OH	H‐DWFKAFYDKVAEKFKEAF‐OH	18

ApoA‐I indicates apolipoprotein A‐I; FAMP, Fukuoka University apoA‐I mimetic peptide. Underlines represent the replaced amino acids from the apoA‐I fragment, apoA‐I (amino acids 196 to 219), amino acids aligned with human apoA‐I.

### FAMP‐Mediated Cellular Cholesterol Efflux in A172 Human Cells and Human and Mouse Macrophages

**Figure 2.**
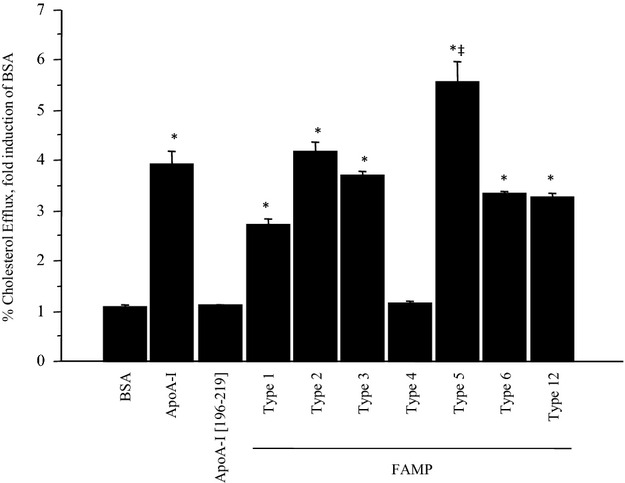
Effects of candidate apoA‐I mimetic peptides on cholesterol efflux in A172 cells. Cellular cholesterol efflux mediated by novel apoA‐I mimetic peptides (FAMPs) were measured in A172 cells. The cells were treated with T0901317 (5 μmol/L) and 9‐*cis*‐retinoic acid (5 μmol/L) in the presence or absence of 20 μg/mL human apoA‐I, apoA‐I fragments, or FAMPs for 4 hours (n=4 to 5 for each group). Values are mean±SD. *P<0.01 vs BSA; ‡*P*<0.01 vs apoA‐I. FAMP indicates Fukuoka University apoA‐I mimetic peptide; apoA‐I, apolipoprotein A‐I; BSA, bovine serum albumin.

**Figure 3.**
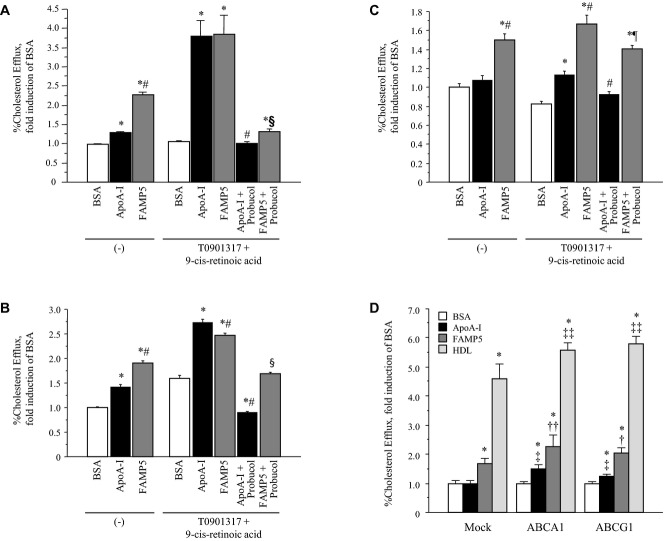
Effects of FAMP5 on cellular cholesterol efflux. ApoA‐I‐ and FAMP5‐mediated cholesterol effluxes were measured in A172 cells (A), mouse peritoneal macrophages (B), and human monocyte‐derived macrophages (C) in the presence or absence of T0901317 (5 μmol/L), 9‐*cis*‐retinoic acid (5 μmol/L), and probucol (10 μmol/L). All experiments were performed for 4 hours in the presence or absence of 20 μg/mL human apoA‐I or FAMP5 (n=4 to 5 for each group). D, COS‐7 cells were transiently transfected with the empty vector (mock) or with human ABCA1 and ABCG1 cDNA, and cholesterol efflux was measured after incubation with 20 μg/mL apoA‐I, FAMP5, or HDL. All experiments were performed for 4 hours (n=6 to 7 for each group). Values are mean±SD. *P<0.01 vs BSA; #*P*<0.01 vs apoA‐I; §*P*<0.01 vs FAMP5; ¶*P*<0.05 vs FAMP5; †*P*<0.05 vs FAMP5 in mock; ††*P*<0.01 vs FAMP5 in mock; ‡*P*<0.01 vs apoA‐I in mock; ‡‡*P*<0.01 vs HDL in mock. FAMP indicates Fukuoka University apoA‐I mimetic peptide; apoA‐I, apolipoprotein A‐I; BSA, bovine serum albumin; ABCA1, ATP‐binding cassette transporter A1; ABCG1, ATP‐binding cassette transporter G1; HDL, high‐density lipoprotein.

FAMP5 showed dose‐dependent induction of cholesterol efflux with saturation effects on a molar basis, and both stimulated efflux by LXR/RXR activation as well as human apoA‐I (Figure 1E). That is, an apoA‐I concentration of 20 μg/mL was apparently adequate for a saturating effect for apoA‐I‐mediated cholesterol efflux. Almost all the novel peptides, except FAMP4, could significantly take up cholesterol and human apoA‐I from human A172 cells treated with LXR/RXR agonists; however, FAMP5 resulted in higher cholesterol efflux from A172 human cells than either human serum apoA‐I, apoA‐I fragments, or other FAMPs (Figures 1B, 2, and 3A).

**Figure 4.**
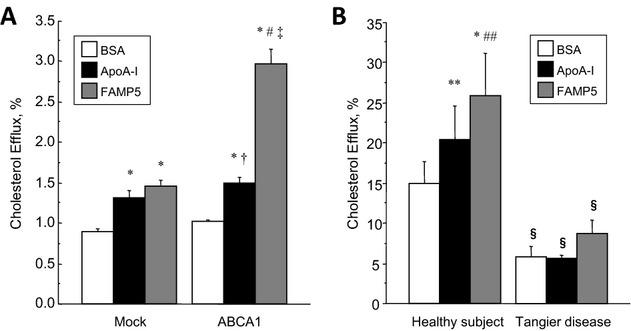
Effects of FAMP5 on cellular cholesterol efflux in ABCA1‐overexpressing and ‐deficient cells. A, Cultivated CHO‐ldlA7 cells were transfected with the empty vector (mock) or human ABCA1 cDNA, and cholesterol efflux was measured (n=6 for each group). All experiments were performed for 4 hours in the presence or absence of 20 μg/mL human apoA‐I or FAMP5. B, Cholesterol efflux was measured in monocyte‐derived macrophages from a healthy subject and a Tangier disease patient. All experiments were performed for 4 hours in the presence or absence of 20 μg/mL human apoA‐I or FAMP5 (n=4 for each group). Values are mean±SD. *P<0.01 vs BSA; **P<0.05 vs BSA; #*P*<0.01 vs apoA‐I; ##*P*<0.05 vs apoA‐I; †*P*<0.05 vs mock; ‡*P*<0.01 vs mock; §*P*<0.01 vs healthy subject. FAMP indicates Fukuoka University apoA‐I mimetic peptide; ABCA1, ATP‐binding cassette transporter A1; CHO, Chinese hamster ovary; apoA‐I, apolipoprotein A‐I; BSA, bovine serum albumin.

**Figure 5.**
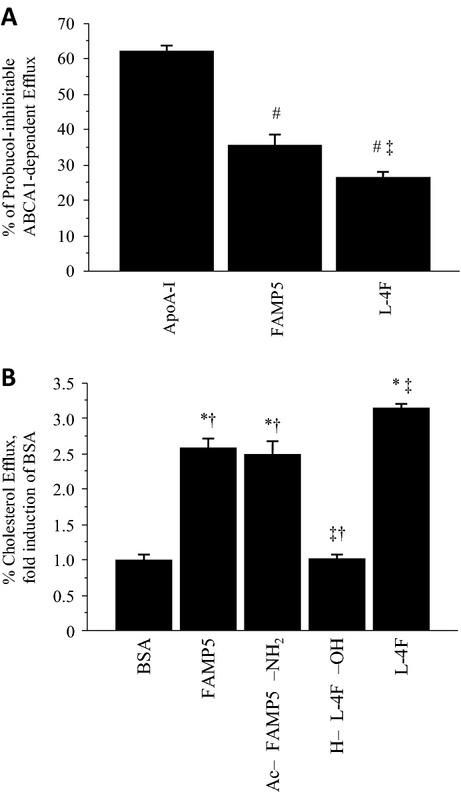
Effects of FAMP5 and other apoA‐I mimetic peptides on cellular cholesterol efflux in A172 human cells. A, Graph represents percent of probucol (10 μmol/L)–inhibitable ABCA1‐dependent cholesterol efflux mediated by apoA‐I, FAMP5, or L‐4F measured in A172 cells. B, Effects of H‐FAMP5‐OH (FAMP5), Ac‐FAMP5‐NH_2_, Ac‐L‐4F‐NH_2_ (L‐4F), and H‐L‐4F‐OH on cellular cholesterol efflux in A172 cells. The experiments were performed for 4 hours in the presence or absence of 20 μg/mL of peptides (n=5 for each group). Values are mean±SD. *P<0.01 vs BSA; #*P*<0.01 vs apoA‐I; ‡*P*<0.01 vs FAMP5; †*P*<0.01 vs L‐4F. FAMP indicates Fukuoka University apoA‐I mimetic peptide; ABCA1, ATP‐binding cassette transporter A1; apoA‐I, apolipoprotein A‐I; BSA, bovine serum albumin.

As in A172 cells, FAMP5 induced cholesterol efflux significantly more than human apoA‐I in both mouse peritoneal macrophages (Figure 3B) and human monocyte‐derived macrophages (Figure 3C). Although there were some differences in the induction of FAMP5‐mediated cholesterol efflux in cells with LXR/RXR activation, the induction strongly suggested that FAMP5 interacted with a membrane cholesterol transporter such as ABCA1. As expected, the ABCA1‐inactivating reagent, probucol, drastically inhibited FAMP5‐ and human apoA‐I‐mediated effluxes (Figure 3A), and transient transfection with *ABCA1* cDNA significantly upregulated FAMP5‐mediated cholesterol efflux in COS‐7 (Figure 3D) and CHO‐ldlA7 (Figure 4A) cells. Moreover, apoA‐I‐ and FAMP5‐mediated cholesterol effluxes were suppressed in circulating blood monocyte‐derived macrophages from an ABCA1‐deficient Tangier disease patient (Figure 4B). Percentage of probucol‐inhibitable, ABCA1‐specific cholesterol efflux and peptide‐mediated total efflux were measured in FAMP5 and another apoA‐I mimetic L‐4F peptide (Figure 5A and 5B). The ABCA1‐specific efflux in FAMP5 was significantly higher than that in L‐4F, although it was significantly lower than human apoA‐I, that is, L‐4F peptide had much more unspecific cholesterol efflux compared with FAMP5 (Figure 5A). In addition, the differences of terminal end modifications in these peptides were considered. L‐4F is an acetylated at its N terminus and amidated at its C terminus, whereas the peptide of the same amino acid sequences does not possess any function for cholesterol efflux, of which has natural amidation and carboxylation at the N terminus and the C terminus, respectively (Figure 5B). In contrast to L‐4F, FAMP5 has no influence by the modification of terminal ends of peptides.

### Pharmacokinetics of ACD‐FAMP in C57BL6 and ApoE^−/−^ Mice

**Figure 6.**
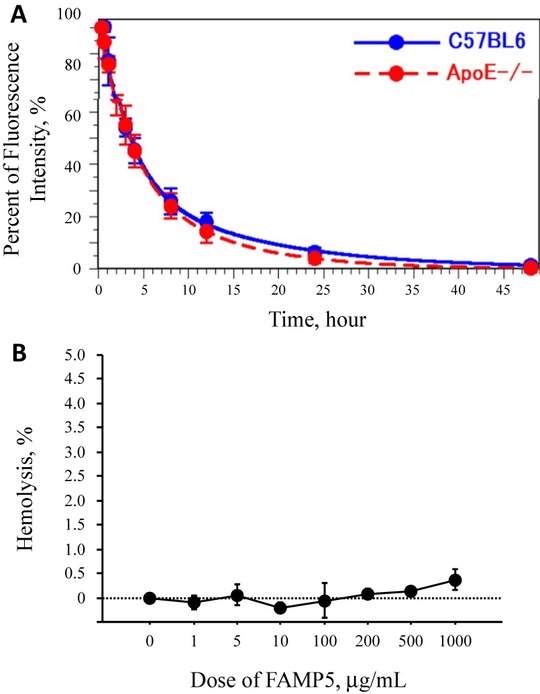
Blood clearance and influence of hemolysis on FAMP5. A, Pharmacokinetics of FAMP5 in C57BL6 and apoE‐deficient mice. Both C57BL6 (n=3) and apoE‐deficient (n=3) mice were intravenously administered 10 mg/kg of fluorescence‐labeled ACD‐FAMP5. Fifteen minutes after ACD‐FAMP5 injection, plasma fluorescence signals peaked and then became attenuated. B, Effect of FAMP5 on red cell hemolysis. Various concentrations of FAMP5 were incubated with red blood cells for 10 minutes at 37°C. Hemolysis was measured as hemoglobin content at an optical density at 540 nm of the supernatant. Hemolysis was expressed as a percentage of the Triton X‐100 lysis. Values are mean±SD. FAMP indicates Fukuoka University apoA‐I mimetic peptide; apoE, apolipoprotein E; ACD, acridone.

Both C57BL6 and apoE‐deficient mice were intravenously administered 10 mg/kg of fluorescently labeled ACD‐FAMP5. Fifteen minutes after ACD‐FAMP5 injection, plasma fluorescent signals peaked and then became attenuated. The fractional catabolic rate (FCR) of plasma ACD‐FAMP5 did not significantly differ between C57BL6 mice (FCR, 0.148±0.024) and apoE‐deficient mice (FCR, 0.173±0.037) (Figure 6A).

### Effect of FAMP5 on Red Cell Hemolysis

FAMP not only elicits cholesterol efflux through specific ABCA1‐mediated pathways but also appears to elicit unspecific cholesterol efflux. Intensive unspecific removal of cholesterol causes lysis. We confirmed the effects of FAMP5‐induced hemolysis using previously established methods (Figure 6B). There were only limited effects of FAMP5 on hemolysis when using a high dosage (up to 1.0 mg/mL).

### Effects of FAMP and ACD‐FAMP on Plasma Lipoprotein Profiles

**Figure 7.**
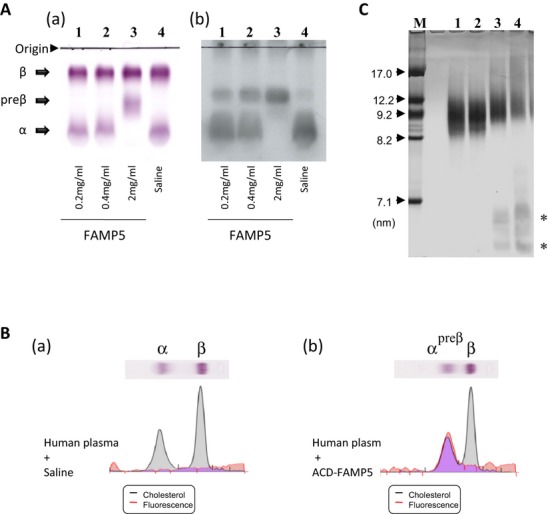
Effects of FAMP on pre‐β HDL formation in vitro. A, FAMP5 (0.2 to 2.0 mg/mL) was incubated with human whole plasma at 37°C for 60 minutes, and the plasma lipoprotein profiles were analyzed by agarose gel electrophoresis (a) and Western blotting with anti‐apoA‐I (b). FAMP5 promoted the migration of apoA‐I to the pre‐β HDL position in a dose‐dependent manner. B, Saline (a) or fluorescence‐labeled ACD‐FAMP5 2.0 mg/mL (b) was incubated with human plasma at 37°C for 60 minutes. A fluorescence signal on the agarose gel under UV light was detected in samples incubated with ACD‐FAMP5. C, FAMP5 was incubated with human HDL (2.22 mg/mL) at 37°C for 60 minutes and subjected to 5% to 20% gradient polyacrylamide gel electrophoresis. After electrophoresis, the gels were stained for proteins with CBB. Incubation with FAMP5 generated small HDL particles (*), such as pre‐β_1_ HDL‐like particles. Lane 1, saline; lane 2, 50 μg/mL FAMP5; lane 3, 250 μg/mL FAMP5; lane 4, 500 μg/mL FAMP5. FAMP indicates Fukuoka University apoA‐I mimetic peptide; apoA‐I, apolipoprotein A‐I; ACD, acridone; HDL, high‐density lipoprotein; CBB, Coomassie Brilliant Blue.

FAMP5 was incubated with human whole plasma for 60 minutes at 37°C, and the plasma lipoprotein profile was analyzed by agarose gel electrophoresis and immunoblotting with anti‐apoA‐I antibody. Incubation with FAMP5 shifted the α HDL band to the pre‐β HDL position (Figure 7A‐a). Western blot analysis with anti‐apoA‐I revealed apoA‐I at the pre‐β HDL position even at low FAMP5 doses (Figure 7A‐b). These observations suggest that incubation of human plasma with FAMP5 transformed α HDL to pre‐β HDL. High doses of FAMP5 completely converted α HDL to pre‐β HDL. Furthermore, incubation of human plasma with ACD‐FAMP5 for 60 minutes at 37°C shifted α HDL to the pre‐β HDL position with fluorescence signals (Figure 7B). Incubation of human HDL with FAMP5 for 60 minutes at 37°C generated pre‐β_1_ HDL‐like small particles on polyacrylamide gel electrophoresis (Figure 7C). The concentration of 500 μg/mL FAMP5 was determined by HDL protein concentration, that is, it was estimated to be approximately 23% and 226%, increasing on a weight and molar basis, respectively, as an apolipoprotein.

### Effects of ACD‐FAMP on Plasma Lipoprotein Profiles in C57BL6 and ApoE^−/−^ Mice

**Figure 8.**
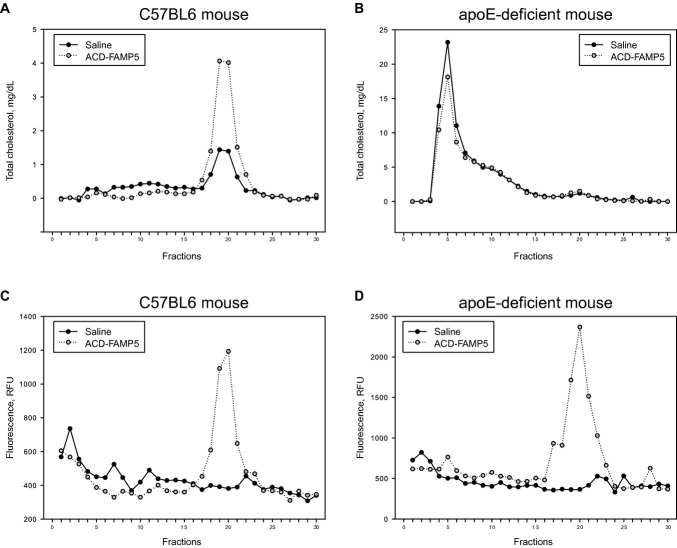
FPLC analyses of lipoprotein profiles in C57BL6 and apoE^−/−^ mice. Mice were intraperitoneally injected with saline (closed circles) or ACD‐FAMP5 (50 mg/kg body weight), and plasma samples were collected after 24 hours. Equal volumes of plasma samples were pooled from C57BL6 (A, C) and ApoE^−/−^ (B, D) mice, and the fractionated lipoproteins were analyzed for cholesterol (A, B) and fluorescent signals (C, D). Fractions 3 to 7 contain VLDL, fractions 8 to 16 contain LDL, and fractions 17 to 24 contain HDL. FPLC indicates fast protein liquid chromatography; FAMP, Fukuoka University apoA‐I mimetic peptide; apoE, apolipoprotein E; ACD, acridone; VLDL, very‐low‐density lipoprotein; LDL, low‐density lipoprotein; HDL, high‐density lipoprotein; RFU, relative fluorescent units.

FPLC analysis was performed on plasma samples collected from C57BL6 and apoE^−/−^ mice 24 hours after injection with FAMP5. Analyses showed that cholesterol was primarily distributed in the HDL fractions in C57BL6 mice (Figure 8A). In contrast, a majority of the cholesterol was present in the low‐density lipoprotein (LDL)– and very‐low‐density lipoprotein–sized fractions in apoE^−/−^ mice (Figure 8B). Interestingly, the fluorescence signals from injection of fluorescent‐labeled ACD‐FAMP5 were detected by FPLC in HDL fractions from both C57BL6 and apoE^−/−^ mice (Figure 8C and 8D). These findings indicated that FAMP5 enters only into HDL particles to form nascent HDL, regardless of whether the particles contain cholesterol.

### Effect of the 16‐Week FAMP Treatment on Atherosclerotic Lesion Formation in ApoE^−/−^ Mice Fed a High‐Fat Diet

**Figure 9.**
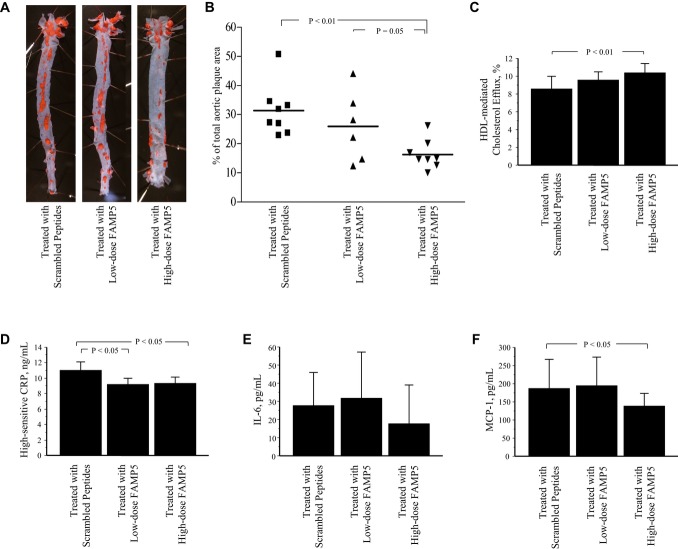
Suppressive effect of FAMP on atherosclerotic lesion formation in apoE^−/−^ mice fed a high‐fat diet. ApoE^−/−^ mice fed a high‐fat diet (0.5% cholesterol and 10% fat) were intraperitoneally treated 3 times per week with scrambled FAMP (n=8), low‐dose FAMP5 (10 mg/kg body weight; n=7), or high‐dose FAMP5 (50 mg/kg body weight; n=8). After 16 weeks, whole aortas were collected and stained with Oil Red O (A). The extent of atherosclerosis is expressed as the percent of the lesion area extending from the ascending aorta to the abdominal bifurcation (B). The biological functioning of HDL was measured as *ex vivo* cholesterol efflux from RAW264 mouse macrophages mediated with HDL collected from FAMP5‐treated apoE^−/−^ mice (C). Plasma high‐sensitive C‐reactive protein (CRP) level (D), plasma interleukin 6 (IL‐6) level (E), and plasma monocyte chemoattractant protein‐1 (MCP‐1) level (F) were measured in apoE^−/−^ mice. Values are mean±SD. FAMP, Fukuoka University apoA‐I mimetic peptide; apoE, apolipoprotein E; HDL, high‐density lipoprotein.

ApoE^−/−^ mice fed a high‐fat diet were treated with low and high doses of FAMP5 for 16 weeks (Figure 9). Long‐term treatment with FAMP5 had no significant effects on plasma total cholesterol, triglyceride, and HDL cholesterol levels by enzymatic assay (Table 2). Surprisingly, the relative surface areas of the plaque lesions were significantly suppressed in the high‐dose FAMP5‐treated group compared with the scrambled‐peptide‐treated group (16.2±5.0% versus 31.3±8.9%, *P*<0.01; Figure 9A and 9B). Plasma lipoprotein profiles were determined using HPLC methods according to established procedures.^34–35^ Each lipoprotein subfraction was assessed for total cholesterol, free cholesterol, triglycerides, and phospholipids by enzymatic assays after separating 20 subfractions based on particle sizes using HPLC. Contrary to our expectations, FAMP5 treatment did not increase plasma HDL cholesterol levels, whereas HPLC analyses showed that free cholesterol level in the small HDL particle‐size subfraction was significantly increased by treatment with high‐dose FAMP5 in apoE^−/−^ mice (Table 3). *Ex vivo* cholesterol efflux mediated by HDL in high‐dose FAMP5‐treated apoE^−/−^ mice showed a significant increase in the biological functioning of HDL (Figure 9C), providing further evidence of changes in the lipid composition of HDL particles. Furthermore, both low and high doses of FAMP equally suppressed plasma high‐sensitive CRP levels in apoE^−/−^ mice (Figure 9D). Other plasma inflammatory markers, IL‐6 and MCP‐1, were also measured in apoE^−/−^ mice treated with FAMP5 for 16 weeks (Figure 9E and 9F). High FAMP doses significantly reduced plasma MCP‐1 levels in the mice as well as reducing CRP.

**Table 2.** Effects of FAMP5 Treatment for 16 Weeks on Plasma Lipid Profiles in High‐Fat‐Fed ApoE‐Deficient Mice

Group	Total Cholesterol, mg/dL	Triglyceride, mg/dL	HDL Cholesterol, mg/dL
Scrambled peptides, n=7	1373.9±499.2	119.9±21.7	12.6±4.8
Low‐dose FAMP5, n=5	1532.8±589.6	120.0±41.6	16.6±4.8
High‐dose FAMP5, n=7	1439.5±271.5	102.7±18.3	12.6±3.7

FAMP indicates Fukuoka University apoA‐I mimetic peptide; apoE, apolipoprotein E. ApoE‐deficient mice were treated intraperitoneally with scrambled peptides, low‐dose FAMP5 (10 mg/kg per day; 3 times a week), or high‐dose FAMP5 (50 mg/kg per day; 3 times a week) for 16 weeks.

**Table 3.** Effects of FAMP Treatment for 16 Weeks on Plasma Lipoprotein Profiles Analyzed by HPLC in High‐Fat‐Fed ApoE‐Deficient Mice

Group	Plasma Lipids	CM (>80 nm), mg/dL	VLDL (30 to 80 nm), mg/dL	LDL (16 to 30 nm), mg/dL	HDL (8 to 16 nm), mg/dL	HDL Subfractions		
						Large Particles	Intermediate Particles	Small Particles
Scrambled peptides, n=7	Cholesterol	106.2±47.8	423.7±96.1	165.3±25.4	23.0±4.5	7.2±1.5	12.0±3.4	3.8±0.5
	Free cholesterol	44.9±20.5	179.8±35.9	80.9±10.1	12.5±1.4	5.7±1.1	5.1±0.5	1.7±0.2
	Triglyceride	13.9±7.5	33.8±7.7	12.3±4.2	3.3±0.8	0.6±0.2	1.5±0.5	1.1±0.3
	Phospholipids	53.0±22.3	228.2±40.0	133.6±20.5	45.5±10.3	12.4±3.3	28.1±7.4	5.0±1.7
Low‐dose FAMP5, n=5	Cholesterol	175.4±40.4*	529.9±135.3	133.1±16.1	22.0±3.7	5.1±0.7*	12.5±3.1	4.4±0.8
	Free cholesterol	70.7±14.4†	220.1±49.0	65.6±6.0†	9.1±0.6*	3.3±0.6*	3.9±0.4*	1.9±0.3
	Triglyceride	26.1±16.8	38.1±19.4	9.5±2.3	2.9±1.2	0.5±0.2	1.5±0.7	0.9±0.5
	Phospholipids	78.4±15.3†	263.8±53.4	94.4±10.4*	29.1±4.9*	5.9±2.1*	21.5±5.4	1.7±1.1*
High‐dose FAMP5, n=7	Cholesterol	173.4±24.0*	509.1±73.8	157.9±32.1	20.3±3.1	5.8±0.6†	10.1±2.5	4.5±0.6
	Free cholesterol	76.6±13.7*	213.8±33.7	75.0±14.1	10.0±1.1*	3.8±0.5*	4.1±0.5*	2.1±0.4†
	Triglyceride	22.1±6.6	35.8±9.7	10.4±2.2	3.7±1.3	0.6±0.1	1.8±0.8	1.3±0.4
	Phospholipids	84.3±14.2*	259.9±44.9	110.6±26.5	23.1±5.2*	6.8±1.0*	15.6±4.9*	0.8±0.3*

FAMP indicates Fukuoka University apoA‐I mimetic peptide; HPLC, high‐performance liquid chromatography; CM, chylomicron; VLDL, very‐low‐density lipoprotein; LDL, low‐density lipoprotein; HDL, high‐density lipoprotein; apoE, apolipoprotein E. ApoE‐deficient mice were treated intraperitoneally with scrambled peptides, low‐dose (10 mg/kg per day, 3 times a week) or high‐dose (50 mg/kg per day, 3 times a week) FAMP type 5 for 16 weeks.

*P<0.01; ^†^*P*<0.05 vs scrambled peptides group.

## Discussion

The aim of this study was to develop short apoA‐I mimetics with the physiological functions of human apoA‐I. Although the mechanism of interaction between ABCA1 and apoA‐I has not been completely resolved, ABCA1 overexpression has been shown to enhance the cellular binding and uptake of apoA‐I.^36–39^ In addition, Chroni et al demonstrated with an apoA‐I deletion mutant that amino acids 220 to 231 along with the carboxyl‐terminal domain of human apoA‐I are important for interaction with ABCA1, which is required for cholesterol efflux and HDL formation.^40^ We synthesized a novel functional apoA‐I mimetic peptide consisting of 24 amino acids and without phospholipids. Previously, several lipid‐free apoA‐I mimetics have been shown to have antiatherosclerotic and antiinflammatory effects in vitro and in animal models, although none of these are available for clinical use. The most important role of apoA‐I is HDL construction, which depends on its amphipathic properties. Furthermore, lipid‐poor or lipid‐free apoA‐I removes free cholesterol from peripheral cells through ABCA1, which is required to form nascent HDL and is a key molecule in the function of apoA‐I in the initial step in the reverse cholesterol transport pathway.^1^ To develop a physiologically active apoA‐I mimetic peptide, we focused on the C terminus (amino acids 196 to 240) of the apoA‐I protein because this region is important for interaction with ABCA1. We synthesized apoA‐I mimetic peptides corresponding to the region around amino acids 220 to 231 of the C terminus of human apoA‐I. Synthetic fragments of 20 to 24 amino acids aligned with human apoA‐I had no significant effects on cellular cholesterol efflux; however, a 31–amino acid fragment did have a significant effect (Figure 1B). Therefore, although this region of apoA‐I is important for interaction with ABCA1, 24 amino acids aligned with human apoA‐I was insufficient to form an α‐helical structure (Figure 1A). In contrast to the 24–amino acid fragments of human apoA‐I, the novel FAMP peptides, except FAMP4, achieved an α‐helical configuration and maintained interaction with ABCA1 despite having only 24 amino acids. As shown by a helical wheel model for FAMP5 in Figure 1C, the typical type of an amphipathic helix is found for apoA‐I, and according to a replacement for 8 of 24 amino acids in an apoA‐I fragment (ApoA‐I [196 to 219]), FAMP5 has acquired this characteristic by having a surface area of its hydrophobic face approximately equal to the size of its hydrophilic face. This balance of the hydrophobic face must be important for lipid interactions, and the hydrophilic face might be important for its ability to cause specific cholesterol efflux.^41^ Of the several FAMPs, FAMP5 was most effective for cholesterol efflux (Figures 1B and 2). Not only did FAMP5 greatly promote cholesterol efflux, our results with ABCA1‐overexpressing cells indicate that it functioned together with the ABCA1 transporter (Figures 3 and 4).

Thus far, several apoA‐I mimetics with α‐helical configurations have been developed, although it is not known whether there are functional differences in the HDL formed through the mediation of these mimetics and whether their activities are ABCA1‐dependent or ‐independent. FAMP5 may play a physiological role similar to apoA‐I, with its efflux function mediated by ABCA1, because the lack of functional ABCA1 transporters blocked FAMP5‐stimulated cholesterol efflux (Figure 3A through 3C). In Tangier disease patients, this results in the accumulation of cholesteryl ester in peripheral tissues such as arteries and peripheral nerves.^28,42^ Thus, FAMP5 is a promising peptide for the suppression of arterial plaque formation in humans.

In contrast to apoA‐I, FAMP5‐mediated efflux was not completely abolished under ABCA1‐inactivated conditions, such as in cells treated with probucol and Tangier macrophages (Figures 3A through 3C and 4B). These results suggest that FAMP5 functions in removing cholesterol through not only the ABCA1 pathway but also another specific pathway that may be dependent on the ABCG1 transporter (Figure 3D). As shown in Figure 3D, COS‐7 cells transiently transfected with the *ABCA1* gene had significantly increased apoA‐I‐ and HDL‐mediated efflux compared with mock transfection. The reason for the high HDL‐mediated efflux in these cells is not clear. However, these cells also had induced apoA‐I‐mediated efflux. This finding suggests that these cells highly express functional ABCA1. As a possible mechanism, HDL might be comparatively rich in small HDL particles or other transporters, and receptors might be activated by ABCA1 overexpression.

FAMP5 had similar potency to other apoA‐I mimetic peptides, such as L37pA and L‐4F. Nevertheless, our peptide was quite small, comprising 24 amino acids, compared with L37pA, although the L‐4F peptide comprises only 18 amino acids. Because FAMP5 was designed to focus on the ABCA1‐binding lesion of human apoA‐I, it acquired ABCA1 specificity for cholesterol efflux, compared with L‐4F. In addition, we have shown that there are differences in the terminal end modifications of these peptides (Figure 5B). L‐4F is acetylated at its N terminus and amidated at its C terminus, which enhances its capacity for cellular cholesterol efflux, whereas the same peptide does not possess any function for cholesterol efflux with natural amidation and carboxylation at the N terminus and the C terminus, respectively. In contrast, peptide FAMP5 had no influence because of modifications of its terminal ends, and these characteristics may allow it to work in an *Escherichia coli* or plant expression system.

FAMP5 has at least 2 possible distinct roles in HDL metabolism, especially pre‐β HDL production. First, FAMP5 enhances the cholesterol efflux mediated by ABCA1‐dependent and ‐independent mechanisms, which leads to the generation of nascent pre‐β HDL particles. Second, incubation of FAMP5 with human HDL or plasma generates small HDL particles and charged apoA‐I‐rich particles, which migrate as pre‐β HDL on agarose gel electrophoresis (Figure 7). Fluorescently labeled ACD‐FAMP5 revealed that FAMP5 could be incorporated into HDL particles but not into LDL and other lipoproteins in vitro and in vivo (Figure 7B and 8). These 2 mechanisms of pre‐β HDL formation cooperate in the antiatherosclerotic function of FAMP5. FAMP5 significantly suppressed aortic plaque formation in apoE^−/−^ mice, although plasma HDL cholesterol levels were unchanged by the 16‐week treatment with FAMP5 (Figure 9). A possible mechanism of the plaque regression might be an improvement of HDL function. The 16‐week treatment with FAMP5 did not increase plasma HDL cholesterol levels and significantly decreased free cholesterol levels of the HDL fraction in apoE^−/−^ mice; however, the free cholesterol levels in the small HDL particle‐size subfraction, which includes pre‐β_1_ HDL particles, was significantly increased by treatment with high doses of FAMP5 (Table 3). The increased free cholesterol levels in the smallest HDL particles indicates an increasing of the pre‐β_1_ HDL particle, and it may lead to enhance HDL‐mediated cholesterol efflux from FAMP5‐treated apoE^−/−^ mice. Although these results may appear contradictory, both human and animal studies over the past decade have suggested that although biological HDL function is known to have cardioprotective effects, quantification of HDL cholesterol levels provides only limited information about those effects.^43–44^ In support of these results, Rader's group has demonstrated that the efflux capacity of serum HDL is a strong predictor of CHD.^45^ Moreover, de la Llera‐Moya et al^11^ showed that the most important predictor of cholesterol efflux from macrophages is not serum HDL cholesterol levels, but rather levels of the small HDL subfraction known as pre‐β_1_ HDL. Thus, pre‐β HDL must be a key molecule for functional HDL. According to a kinetic study, the conversion of nascent pre‐β HDL to mature α HDL is 10 times faster than the generation of pre‐β HDL from α HDL.^46^ On the other hands, FAMP5 takes up cholesterol from peripheral cells, which results in the formation of nascent pre‐β HDL particles, and, in parallel, FAMP5 accelerates the generation of pre‐β HDL, which is converted from mature HDL. Finally, FAMP5 may function by directly removing cholesterol from macrophages, increasing HDL neoformation and HDL biological function, and augmenting antiinflammatory effects for aortic plaques in apoE^−/−^ mice.

In conclusion, a newly developed apoA‐I mimetic peptide, FAMP5, significantly suppresses aortic plaque formation through the enhancement of the biological functioning of HDL in apoE^−/−^ mice fed a high‐fat diet. Thus, FAMP5 may have tremendous atheroprotective potential and prove to be a new therapeutic tool for cardiovascular disease.
